# Incidental Finding of an Injured Anomalous Artery in the Forearm: To Repair or Not

**DOI:** 10.1055/s-0044-1801837

**Published:** 2025-01-21

**Authors:** G.I. Nambi

**Affiliations:** 1 Plastic, Burns, Hand & Microsurgical Services, Kavin Medical Center, Erode, Tamil Nadu, India

Anomalous vessels are uncommon findings during the course of surgical procedures and even rarer are the injured anomalous vessels presenting an on-table dilemma to the surgeon of whether to repair or ligate.

**Fig. 1:**
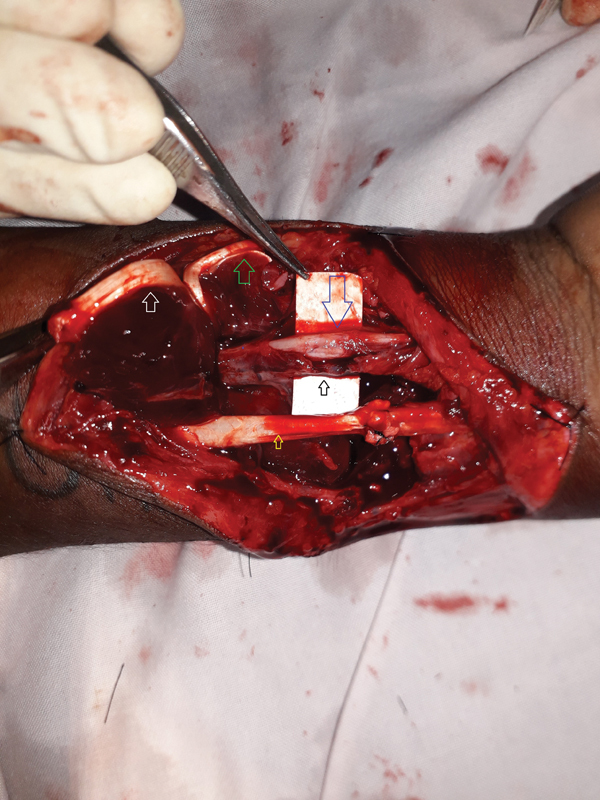
Partially injured anomalous vessel ( *black small arrow* ) overlying the median nerve ( *blue large arrow* ). In this case, it was the presence of the persistent median artery that saved the median nerve from injury. The repaired flexor carpi radialis tendon ( *yellow arrow* ) is on the lateral side. The palmaris longus tendon ( *green arrow* ) and the flexor digitorum superficialis tendon of the index finger ( *white arrow* ) are reflected on the medial side.

**Fig. 2:**
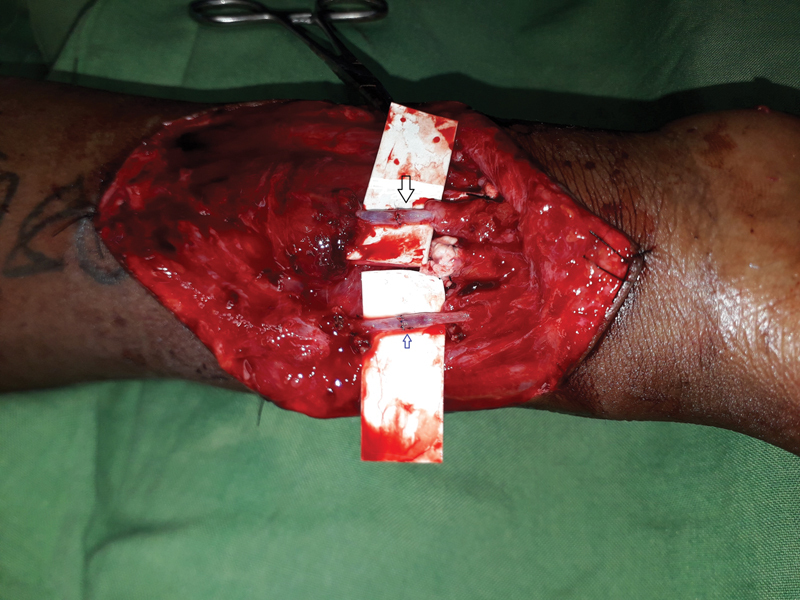
Microsurgical repair of both the radial artery ( *blue small arrow* ) and the persistent median artery ( *black large arrow* ). In this image, the median nerve is hidden under the background material.

**Fig. 3:**
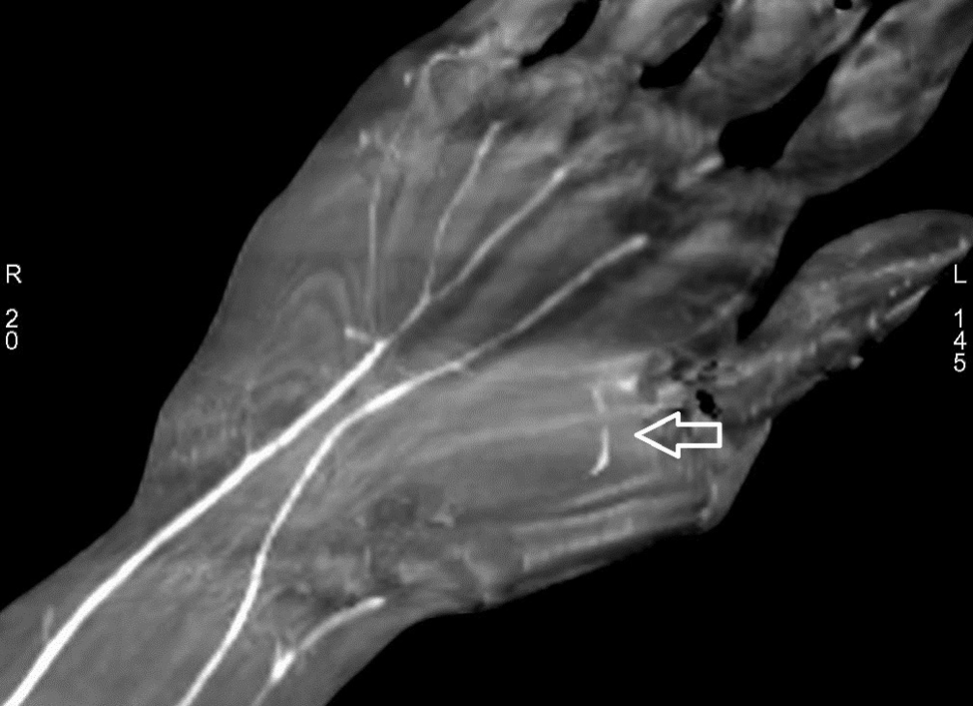
The ulnar artery supplying the little finger, ring finger, and ulnar side of the middle finger, the PMA supplying the radial side of the middle finger and both sides of the index finger, and the radial artery supplying the thumb. A rudimentary superficial palmar arch ( *white arrow* ) between the radial digital artery of the index finger and the ulnar digital artery of the thumb.

A 19-year-old man who had sustained glass cut injury to his right distal forearm was explored 4 hours later, for surgical repair under tourniquet control and supraclavicular block. The palmaris longus, long flexors of the thumb, index and middle fingers, flexor carpi radialis, and radial artery were found to be divided. After repairing the injured deeper tendons, the divided ends of the radial artery were applied with micro-clamps and the tourniquet was released. After this, severe bleeding was noted in the operative field, which led to reinflation of the tourniquet and reassessment of the operative field. The source of the bleeding was found to be a partially injured persistent median artery (PMA) overlying the median nerve ( Fig. 1 ). A decision was made to repair this along with the injured radial artery and both were repaired ( Fig. 2 ). The postoperative period was uneventful and 3 months later, computed tomography angiogram (CTA) study of the vascularity of the limb revealed the ulnar artery supplying the little finger, ring finger, and ulnar side of the middle finger, the PMA supplying the radial side of the middle finger, index finger, and radial artery supplying the thumb. A rudimentary superficial palmar arch (SPA) was found between the radial digital artery of the index finger and ulnar digital artery of the thumb ( Fig. 3 ) and CTA of the opposite upper limb was refused by the patient.

**Fig. 4:**
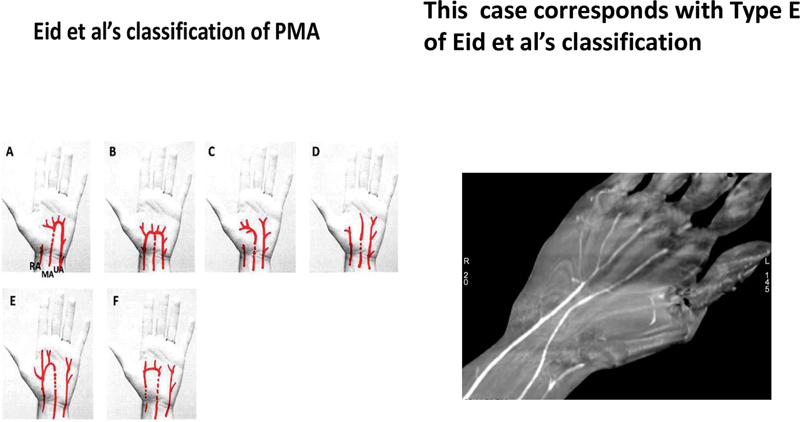
Image of Eid et al's types and this case corresponds to type E. PMA, persistent median artery.

PMA 1 is seen in 11.53% of Indians 2 and could be an ante brachial type, which is partially involuted, or a palmar type, which is larger, longer, and reaches to the hand. The palmar type can be associated with anatomical variations 2 such as the bifid median nerve and may contribute to the vascularity of the hand by forming a part of the SPA, may not form a superficial arch, and rather give rise to common digital arteries directly. Tsagarakis et al 3 inferred that an injured PMA should be repaired owing to their contribution toward the blood supply of the hand. Eid et al 4 studied extensively about the PMA, radial and ulnar arteries, and their contribution to digital vascularity and SPA. They found that the main patterns were type A (median and ulnar) and type B (radial, median, and ulnar). The SPA was complete in types A and B, incomplete in types E and F, and there was no SPA in types C and D. In this case, the decision to repair the injured PMA was prudent as revealed by CTA. The PMA contributed significantly towards digital vascularity and was of type E ( Fig. 4 ) as per the classification of Eid et al. 4 To conclude, it is safer to repair any aberrant vessels in the distal limbs than to ligate as ligation could compromise the distal limb or digital vascularity.
